# Personality styles in patients with fibromyalgia, major depression and healthy controls

**DOI:** 10.1186/1744-859X-6-9

**Published:** 2007-03-09

**Authors:** Hans M Nordahl, Tore C Stiles

**Affiliations:** 1 Department of Psychology, NTNU, 7491 Trondheim, Norway; 2 Department of Psychiatry and Behavioural Medicine, NTNU, Box 3008, 7442 Trondheim, Norway

## Abstract

**Background:**

The fibromyalgia syndrome (FMS) is suggested to be a manifestation of depression or affective spectrum disorder. We measured the cognitive style of patients with FMS to assess personality styles in 44 patients with fibromyalgia syndrome (FMS) by comparing them with 43 patients with major depressive disorder (MDD) and 41 healthy controls (HC).

**Methods:**

Personality styles were measured by the Sociotropy and Autonomy Scale (SAS) and the Dysfunctional Attitude Scale (DAS). The Structured Clinical interview for DSM Axis I was applied to Axis I disorders, while the Beck Depression Inventory was used to measure depression severity.

**Results:**

Patients with FMS in general have a sociotropic personality style similar to patients with MDD, and different from HC, but FMS patients without a lifetime history of MDD had a cognitive personality style different from patients with MDD and similar to HC.

**Conclusion:**

These findings suggest that a depressotypic personality style is related to depressive disorder, but not to FMS.

## Background

Fibromyalgia is characterized by symptoms of chronic widespread pain and stiffness, multiple tender points at specific anatomical sites, chronic fatigue and sleep disturbance [1-3]. Its aetiology remains unknown, although some biological mechanisms have been identified [4,5]. Since fibromyalgia resembles many psychiatric disorders in that it lacks solid evidence of recognizable anatomical alterations, it has been suggested to be a manifestation of hysteria [6], depression [7] or affective spectrum disorder [8]. Others have, however, asserted that fibromyalgia is not a psychiatric disorder [9], and that fibromyalgia develop as response to an overactive lifestyle [10] or in the absence of psychological factors [11].

Four studies have examined the occurrence of DSM syndromal disorders in patients with primary fibromyalgia and rheumatoid arthritis [7,12-14]. Two studies found a significantly higher occurrence of a lifetime diagnosis of depressive disorder in fibromyalgia patients than in arthritis patients [7,13], while the other two did not find group differences in the occurrence of any DSM syndromal disorders [12,14]. In addition two studies have compared the frequency of lifetime psychiatric disorders in fibromyalgia patients to subjects without a pain syndrome [12,15]. One of them found that fibromyalgia patients from a tertiary care setting, but not community fibromyalgia residents who had not sought medical care for their symptoms, were significantly more often assigned a depressive disorder, anxiety disorder and somatization disorder compared with healthy controls.

Studies using the Minnesota Multiphasic Personality Inventory [16-18] or a similar personality test [19] have shown psychological abnormalities in patients with FMS seen in rheumatological clinics, compared with patients with rheumatoid arthritis and normal controls.

Since fibromyalgia is associated with mood disorders and it has been suggested that FMS is a manifestation of depression or affective spectrum disorder, it is important to assess the cognitive style of patients with FMS because more recent theories [20] have suggested that certain types of cognitions may play a major role in the aetiology, maintenance, and treatment of clinical depression. Beck for example has proposed that cognitions such as dysfunctional attitudes or clusters of depressogenic schemas are trait-like attributes that render individuals vulnerable to depression [20].

Several studies have shown that dysfunctional attitudes and sociotropy, but seldom autonomy, are salient in depressed patients, although it is unclear whether these cognitions are related to depressive disorder, syndrome depression or psychiatric disorder in general [21]. However, no studies have so far specifically examined to what extent these cognitions are typical of patients with FMS.

Thus the purpose of the present study was to assess these cognitions in patients with FMS. Since it has been suggested that FMS is intrinsically related to depression, a group of psychiatric outpatients with major depressive disorder (MDD) was included as a comparison group. In addition, a group of healthy controls (HC) was included to assess normal values.

## Methods

### Subjects

The study consisted of three groups of subjects: 44 patients with FMS, 43 patients with MDD and 41 HC. The patients with FMS were recruited from the local association of fibromyalgia, while the patients with major depression were mainly selected from patients referred to our general psychiatric outpatient clinic. The HC were recruited from the general population living in the same area as the two patient groups.

The fibromyalgia patients underwent a detailed medical history and a thorough clinical examination by a physician at the Department of Physical medicine at the University Hospital. To be included in the study they had to meet the diagnostic criteria of both Smythe [1] and Yunus et al [2]. Retrospective investigation of the patients' data revealed that all patients also fulfilled the American College of Rheumatology 1990 (ARC-90) criteria [3]. The patients with MDD were included if they met the criteria for a unipolar, nonpsychotic major depressive disorder according to the Structured Clinical interview for a DSM Axis I disorder (SCID-I)[22,23] and were free of acute and chronic medical disorders. The HC were included if they reported no history of psychiatric disorder, reported no distribution of pain intensity and scored nine or lower on the Beck Depression Inventory [24] indicating normal mood [25].

### Psychiatric diagnoses

All patients were diagnosed using the Structured Clinical Interview for Axis I disorders [22,23] conducted by an experienced clinical psychologist. The inter-rater reliability was assessed by using a paired-rater design. Videotaped interviews of a diagnostically mixed group of 20 patients were randomly drawn and subsequently observed and rated by another clinical psychologist. Kappa values for Major depressive disorder was 0.92.

### Depression severity

The Beck Depression Inventory [24] is a 21-item self-report inventory, which extensively has been shown to be a reliable and valid measure of syndrome depression severity in both clinical and non-clinical populations [25].

### Cognitive personality measures

The Dysfunctional Attitudes Scale [26] is a self-report inventory consisting of 40-items designed to measure underlying cognitions that predispose individuals vulnerable for developing depression. Scores range from 40–280, and subjects rate the degree of agreement with each statement on a 7 point Likert scale. Examples of statements include "if I do not do well all of the time, people won't respect me", and "if I fail at work, then I am a failure as a person". Test-retest reliability of two and three months periods are acceptable [27] and validity is evidenced by Hamilton and Abrahamson [28] who reported that depressed patients were observed to have higher DAS scores than both non-depressed patients and healthy controls.

The Sociotropy-Autonomy Scale (SAS) [29] is a 60-item self-report inventory, which measures two stable, independent dimensions of cognitive personality traits called sociotropy and autonomy. Sociotropy refers to dependent traits, characterized by an intense need for love, approval and being esteemed by others. Autonomy is defined as an excessive personal demand for accomplishment and freedom from control by others. Thirty items assess sociotropy and thirty items assess autonomy, and the respondents indicates on a 5-point Likert scale the percentage of time each of the statements describe the respondents thinking and behaviour. Examples of SAS-statements are: "I find it difficult to be separated from people I love" (sociotropy) or "it is important for me to be free and independent" (autonomy). The internal reliabilities of sociotropy and autonomy have been high as indicated by Chronbach alfas of .90 and .83, respectively [29]. The test-retest reliabilities over 10 weeks were .80 for sociotropy and .76 for autonomy in student samples [30]. The sociotropy scale has demonstrated good convergent validity with other measures of dependency and affiliation [31], but convergent validity seems to be more inconsistent for the autonomy scale [32].

### Statistical analysis

Chi-square tests were used to examine possible group differences in the distribution of gender as well as psychiatric diagnoses. Mann Whitney U-tests were conducted to examine potential differences in symptom duration between the two patient groups. Analyses of variance (ANOVAs) were performed to examine group differences in age and depression severity. Overall analyses of covariance (ANCOVAs) were used to analyse the various cognitive personality measures. Age was used as a covariate. ANCOVAs with a significant main effect were followed up with two-group ANCOVAs. P values were considered statistical significant if p < .05.

## Results

### Demographic and clinical variables

Table 1 presents the demographic and clinical characteristics of the three subjects groups. ANOVA indicated a significant overall group difference in age (F(2,121) = 5.34, p < 0.001). The patients with FMS were significantly older than the patients with MDD (F(1,85) = 16.27, p < 0.001) and the HC (F(1,78) = 9.73, p < 0.01). There were, however, no significant sex differences between groups (χ^2 ^(2) = 5.78, p < 0.05) and the two patient groups did not differ in symptom duration (Z = 1.39, p < 0.05).

**Table 1:** Demographic and clinical characteristics of the three subject groups.

	**Fibromyalgia**		**Major depression**	**Healthy controls**
	Total (N = 44)	Nondepressed (N = 19)	(N = 43)	(N = 41)
	**M (SD)**	**M (SD)**	**M (SD)**	**M (SD)**
Age (years)	47.3 (12.6)	47.9 (10.9)	38.1 (9.6)	37.8 (11.2)
Symptom duration	13.2 (10.6)	12.9 (9.8)	10.4^a ^(8.9)	0.0 (0.0)
Beck Depression				
Inventory	14.9 (10.1)	10.9 (8.0)	24.1 (6.5)	2.3 (2.6)

	**N (%)**	**N (%)**	**N (%)**	**N (%)**
Female gender	41 (93.2)	17 (89.5)	29 (67.4)	27 (73.0)

Note: a = symptom duration was calculated on the basis of the first onset of a major depressive episode.

As expected, ANOVA indicated a significant overall group difference in depression severity (F(2,121) = 5.26, p < 0.01). Follow up analyses revealed that the patients with MDD were significantly more depressed than both the patients with FMS (F(1,85) = 4.91, p < 0.01) and the HC (F(1,82) = 12.92, p < 0.001), while the patients with FMS were significantly more depressed than the HC (F(1,83) = 14.94, p < 0.001). The patients with FMS and MDD did not differ in the frequency of Generalised anxiety disorder (χ^2 ^(1) = 0.2, ns) or Somatoform disorder (χ^2 ^(1) = 1.1, ns).

### Cognitive personality measures

The means and standard deviations of the various cognitive personality measures for the three groups are presented in table 2.

**Table 2:** Means and standard deviations of the cognitive personality measures for the three subject groups.

	**Fibromyalgia**		**Major depression**	**Healthy controls**
	Total (N = 44)	Nondepressed (N = 19)	(N = 43)	(N = 41)
Cognitive measures:	M (SD)	M (SD)	M (SD)	M (SD)
Sociotropy	64.8^a ^(16.5)	53.3^b ^(14.7)	73.4^a ^(16.6)	57.6 (15.7)
Autonomy	65.4 (14.3)	62.6 (15.1)	62.7 (16.3)	66.8 (13.6)
Dysfunctional				
Attitudes	107.9^b ^(19.8)	95.3^b ^(18.3)	124.2^a ^(31.2)	99.8 (20.3)

Note. a = p < .05 compared to healthy controls. b = p < .05 compared to major depressed patients.

ANCOVAs with age as covariate indicated that the patients with MDD had significantly higher sociotropic and DAS scores compared to the HC (F(1,82) = 7.21, p < 0.01 and F(1,82) = 5.14, p < 0.05, respectively) and significantly higher DAS scores compared to the patients with FMS (F(1,85) = 3.4, p < 0.05), while the patients with FMS had significantly higher sociotropic scores compared to the HC (F(1,82) = 3.23, p < 0.05). The autonomy scores did not differ between groups. Since the sex distribution was different in the three groups, the three group ANCOVA was repeated by including only the female subjects. The results remained virtually the same, thereby ruling out the potential confounding role of sex.

Since a substantial proportion of the patients with FMS met the criteria for a lifetime history of MDD according to the SCID interview, it was possible that the scores on the cognitive personality measures were confounded by high prevalence of depressive disorder among the patients with FMS. To test this possibility the patients with FMS were subdivided into three subgroups, one consisting of those with a concurrent MDD (N = 13), and one with those without such a history (N = 19) and one with those without a concurrent MDD but with a previous history of MDD (N = 12). ANOVA indicated that the FMS patients without a lifetime history of MDD were significantly less depressed as measured on the Beck Depression Inventory (BDI) both those with a concurrent MDD (F(2,46) = 4.15, p < 0.01) and those with a previous history of MDD (F(2,46) = 1.99, p < 0.05).

Moreover, an ANCOVA with age as a covariate indicated that the FMS patients without a lifetime history of MDD had significantly lower scores on sociotropy than both FMS patients with a concurrent MDD (F(2,44) = 3.06, p < 0.01) and FMS patients with a previous history of MDD (F(2,45) = 3.04, p < 0.01). In addition, FMS patients without a lifetime history of MDD scored significantly lower on the DAS than both FMS patients with a concurrent MDD (F (2,36) = 2.73, p < 0.05) and FMS patients with previous history of MDD (F 2,36) = 2.61, p < 0.05).

ANCOVA with age as a covariate indicated that the patients without a lifetime history of MDD did not differ from the HC on any of the cognitive personality measures, but they had significantly lower sociotropic (F(1,54) = 4.81, p < 0.01) and DAS (F(1,54) = 4.40, p < 0.05) scores compared to the patients with MDD.

## Discussion

We found that the patients with MDD had significantly higher sociotropic and DAS scores, but not higher autonomy scores, compared to the HC. This is consistent with earlier studies [21] and partially in accordance with Beck's model of depression [20]. The patients with FMS had significantly lower level of dysfunctional attitudes compared to the patients with MDD, and significantly higher sociotropic scores compared to the HC. However, when the effects of both a concurrent and lifetime MDD were controlled for, by only including the patients without a lifetime MDD in the statistical analyses, then it was clearly demonstrated that the patients with FMS have a cognitive personality style which is similar to HC, but significantly different from patients with MDD.

Moreover, FMS patients without a lifetime history of MDD exhibited significantly higher levels of sociotropy and dysfunctional attitudes than both FMS patients with concurrent or previous history of FMS. The results suggest that a cognitive personality style characterised by high sociotropic traits and a high level of dysfunctional attitudes is typical of patients with MDD, but not typical of patients with FMS unless they meet the criteria for a concurrent or lifetime MDD. This is turn contradicts the view that FMS is a variant of depressive disorder [7] or an affective spectrum disorder [8]. The present study shows that although the prevalence of depression in patients with FMS is relatively high, cognitive personality styles related to depression are not necessary a part of the FMS. This is also consistent with another recent study showing that concurrent depressive disorder and FMS may be independent, but that the effect of the cognitive appraisals of the FMS symptoms may induce depression in the FMS patients [9].

A contrasting view draws on a recent approach based on a family and gene polymorphism studies. These studies have provided evidence that both mood disorders and enhanced pain sensitivity are found more frequently among the first degree relatives of persons with FMS compared to persons with rheumatoid arthritis or healthy persons [33]. Similarly, other studies have demonstrated greater frequencies of gene polymorphisms in the regulatory region of the 5-HTT gene among patients with FMS compared to healthy controls [34]. This specific polymorphism is also associated with both MDD and various anxiety disorders. Although this link is not very strong, and observed primarily among women, the link between FMS and MDD may be mediated by genetic or biological factors.

Some limitations of the present study must be considered. First, the patient selection procedures used in the present study may have led to biased sampling of patients, which in turn may limit the generalizability of the results. Since the patients with fibromyalgia were recruited from a member association, it is possible that the sample was biased towards increased psychiatric pathology compared with patients referred to rheumatology clinics and subjects with fibromyalgia who do not seek medical care [15]. On the other hand, it has been suggested that patients with fibromyalgia who have psychological problems are more likely to be seen at a rheumatology clinic because of referral bias [35]. It is also noteworthy that none of the patients with fibromyalgia were currently in psychiatric treatment, and only a minority of patients with fibromyalgia (18%) had ever sought help. Second, it should be borne in mind that the reliability of one-time assessment of lifetime psychiatric disorder has been reported to be moderate only [36]. Third, although our data seems to be internally valid and well controlled based on the phenotypic cognitive style of depressed patients and patients with FMS, these recent studies does not rule out the possibility of a biologically mediated relationship between FMS and MDD, indicating that there could be a relationship on the genotypic level.

## Conclusion

There has been a large amount of views and research about the classification of FMS in the last decades. A central issue has concerned to what extent FMS is a form of depression or an affective spectrum disorder. A resolution of this issue is central to both the development of methods and for the understanding of the aetiological processes that underlie FMS. Although there might be a common gene polymorphism in FMS and Major depression, our results indicate that these disorders differ with regard to depressogenic personality style and that major depression in patients with FMS occurs primarily as a sequel to fibromyalgia.

## Competing interests

The author(s) declare that they have no competing interests.

## Authors' contributions

The authors contributed equally to the manuscript, and both have been involved in drafting of the manuscript and given the final approval of the submitted version.
